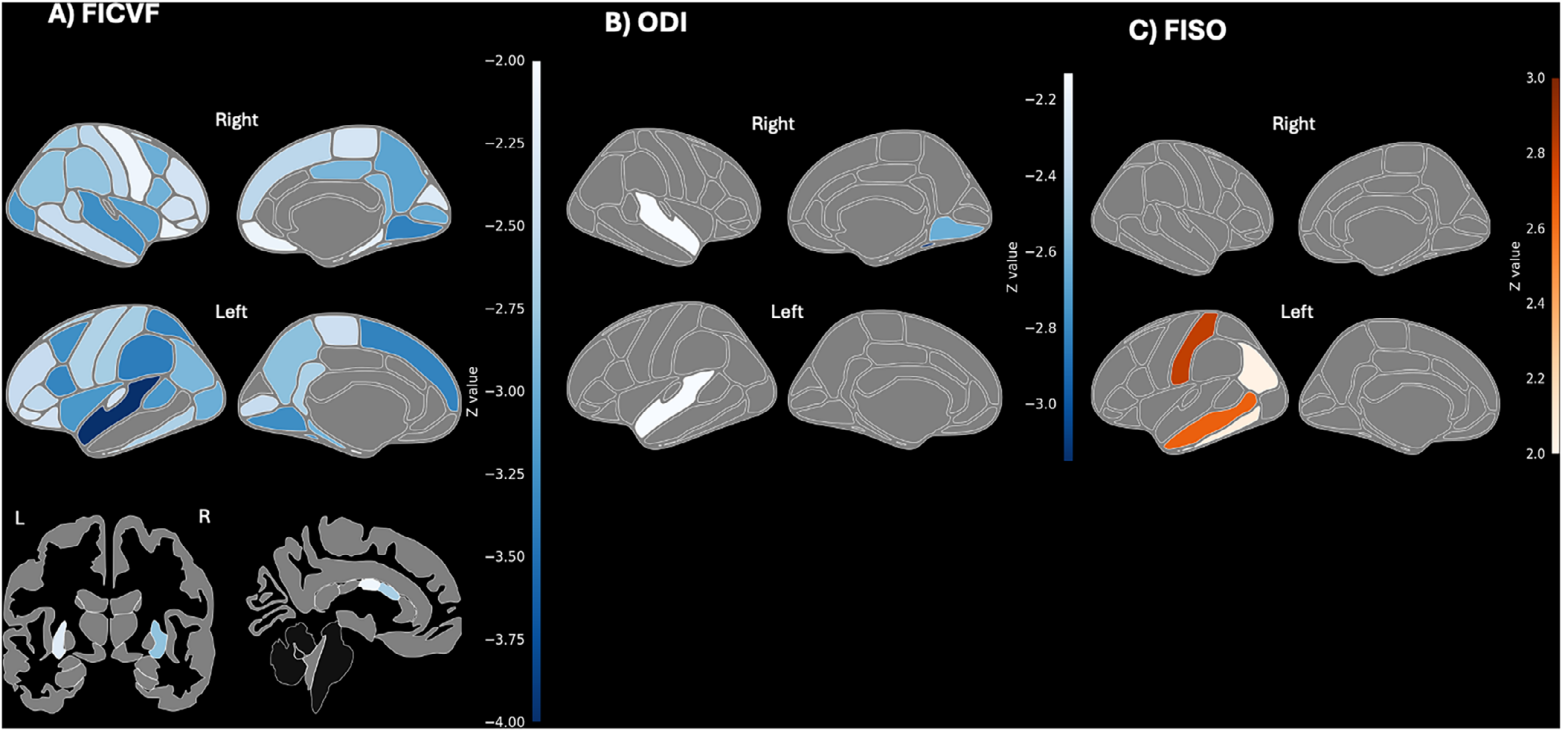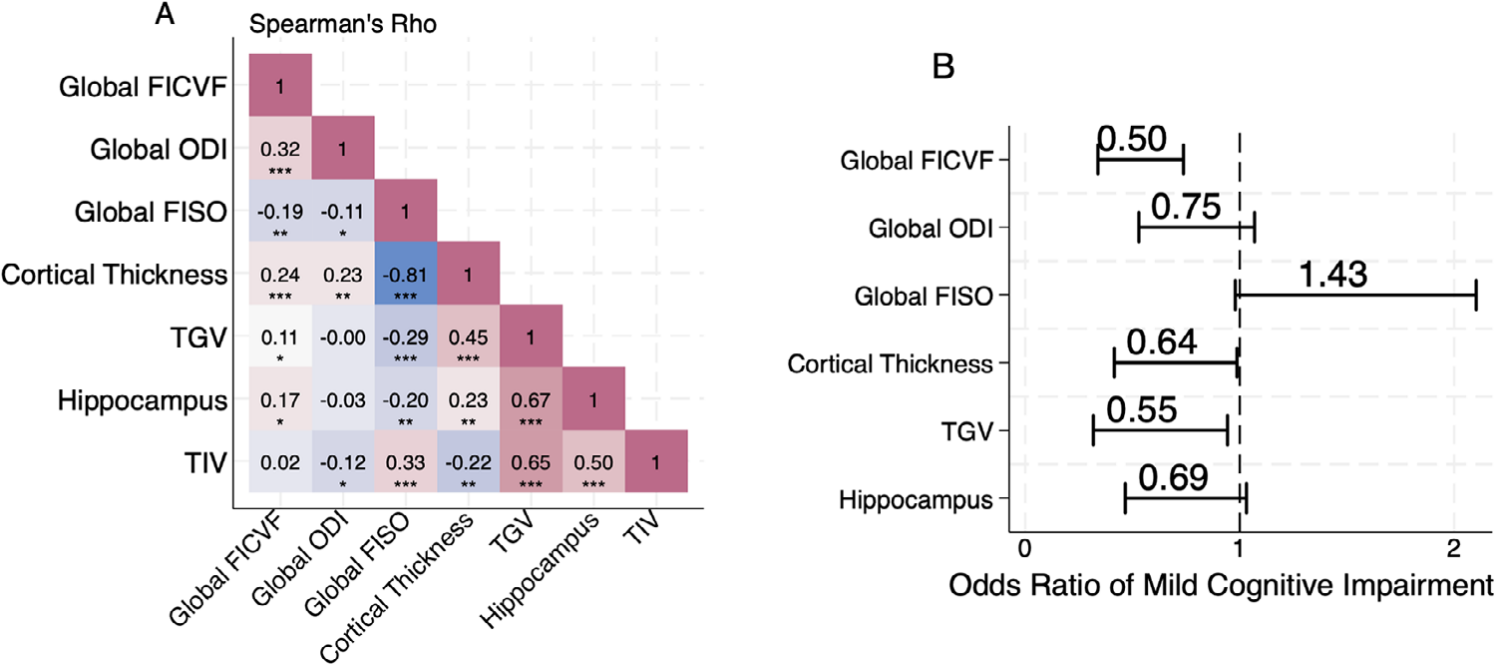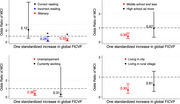# Associations Between Brain Microstructure, Cognition and Socioeconomic Factors in a Diverse Cohort : the LASI‐DAD Study

**DOI:** 10.1002/alz70856_099412

**Published:** 2025-12-25

**Authors:** Yingxu Liu, Kirsten M Lynch, Pranali Khobragade, Joyita Banerjee, Niranjan Khandelwal, Jinkook Lee, Leon M. Aksman

**Affiliations:** ^1^ Mark and Mary Stevens Neuroimaging and Informatics Institute, University of Southern California, Los Angeles, CA, USA; ^2^ Center for Economic and Social Research, University of Southern California, Los Angeles, CA, USA; ^3^ All India Institute of Medical Sciences, New Delhi, India; ^4^ National Institute for Medical Sciences and Research Centre, Jaipur, Rajasthan, India

## Abstract

**Background:**

Superficial white matter microstructural changes may precede widespread macro‐structural atrophy in grey matter and cognitive impairment. However, understanding such relations in older, non‐Western populations is limited; moreover, whether microstructure‐cognition relations differ across disadvantaged socioeconomic backgrounds is unclear.

**Method:**

Our analytic sample included 193 older adults (69.0 years ± 5.0) with multi‐shell diffusion MRI acquisitions from Wave 2 of the Longitudinal Aging Study in India – Diagnostic Assessment of Dementia (LASI‐DAD; collected 2022‐2024; Table 1). Regional and global NODDI metrics [intracellular volume fraction (FICVF), orientation dispersion index (ODI), and isotropic free water volume (FISO)] were derived from white matter underlying the cortex. Logistic regression models assessed regional NODDI metrics in relation to mild cognitive impairment (MCI), defined as a Clinical Dementia Rating (CDR) score of 0.5 and above, adjusting for age, sex, and head motion. Global NODDI metrics (averaged across cortical regions) were compared with FreeSurfer‐based macrostructural measures, including total grey matter volume (TGV), cortical thickness, hippocampal volume, and total intracranial volume (TIV). Interaction effects of socioeconomic factors, including literacy (illiterate, literate with accurate reading, literate with reading errors), education (below or above high school), employment (working or not), and community setting (urban vs. rural), on the NODDI‐cognition relationship were examined.

**Result:**

Higher FICVF in a wide range of cortical regions was associated with lower risk of MCI, with FICVF showing greater sensitivity than ODI and FISO (Figure 1). Stronger effects were observed in the temporal and parietal lobes, subcortex, and middle frontal regions.

Global FICVF also outperformed other NODDI metrics and macrostructural measures in detecting MCI (Figure 2).

Additionally, literacy significantly modify the FICVF‐cognition relationship (Figure 3). Similar interaction trends, though nonsignificant, were noted in education, employment and community settings.

**Conclusion:**

These findings support the role of neurite density, as measured by FICVF, in predicting cognitive outcomes in community‐based populations. Interestingly, a stronger FICVF‐cognition relationship was observed among individuals with disadvantaged socioeconomic backgrounds. Specifically, lower risks of MCI associated with increased FICVF were seen in those who are illiterate, literate with reading errors, have lower education, are unemployed, or reside in urban areas, compared to their counterparts.